# MOBILIZING NETWORKS TO ADDRESS DEMENTIA IN A LATINO COMMUNITY

**DOI:** 10.1093/geroni/igz038.2735

**Published:** 2019-11-08

**Authors:** Caroline Gelman, Nancy Giunta

**Affiliations:** 1 Silberman School of Social Work, New York, United States; 2 Silberman School of social Work, Hunter College, CUNY, New York, New York, United States

## Abstract

Many Latino older adults delay seeking help for symptoms of Alzheimer’s Disease or Related Dementia (ADRD) due to substantial barriers to services. Community-based Natural helpers (NHs) can increase health-related knowledge and can serve as full partners in health education and promotion. This paper presents the process and product of the first phase of a community-based participatory research study to develop a culturally-tailored intervention increasing knowledge about ADRD and services in East Harlem, NY. We describe the results of the initial survey and development of El Barrio SHARE, an intervention that recruits and trains community residents to provide information and referrals about dementia, tapping into natural community networks of people (hairdressers, bodega clerks, mail carriers) who interact with and have longstanding relationships with older adults in the course of their work. NHs are well-positioned to observe and detect problems, and can link elders to relevant, culturally-sensitive resources, accessible support, and treatment.

